# Domestic Animals and Epidemiology of Visceral Leishmaniasis, Nepal

**DOI:** 10.3201/eid1602.090623

**Published:** 2010-02

**Authors:** Narayan Raj Bhattarai, Gert Van der Auwera, Suman Rijal, Albert Picado, Niko Speybroeck, Basudha Khanal, Simonne De Doncker, Murari Lal Das, Bart Ostyn, Clive Davies, Marc Coosemans, Dirk Berkvens, Marleen Boelaert, Jean-Claude Dujardin

**Affiliations:** B.P. Koirala Institute of Health Sciences, Dharan, Nepal (N.R. Bhattarai, S. Rijal, B. Khanal, M.L. Das); Institute of Tropical Medicine, Antwerp, Belgium (N.R. Bhattarai, G. Van der Auwera, N. Speybroeck, S. De Doncker, B. Ostyn, M. Coosemans, D. Berkvens, M. Boelaert, J.-C. Dujardin); London School of Hygiene and Tropical Medicine, London, UK (A. Picado, C. Davies); Université Catholique de Louvain, Brussels, Belgium (N.R. Bhattarai); University of Antwerp, Antwerp (M. Coosemans, J.-C. Dujardin); Prins Leopold Institute, Antwerp (J.-C. Dujardin); 1 Deceased. This article is dedicated to his memory.

**Keywords:** Visceral leishmaniasis, *Leishmania donovani*, transmission, epidemiology, reservoir host, goat, Nepal, parasites, vector-borne infections, research

## Abstract

On the Indian subcontinent, visceral leishmaniasis (VL) is considered an anthroponosis. To determine possible reasons for its persistence during interepidemic periods, we mapped *Leishmania* infections among healthy persons and animals in an area of active VL transmission in Nepal. During 4 months (September 2007–February 2008), blood was collected from persons, goats, cows, and buffaloes in 1 village. *Leishmania* infections were determined by using PCR. We found infections among persons (6.1%), cows (5%), buffaloes (4%), and goats (16%). Data were georeferenced and entered into a geographic information system. The bivariate K-function results indicated spatial clustering of *Leishmania* spp.–positive persons and domestic animals. Classification tree analysis determined that among several possible risk factors for *Leishmania* infection among persons, proximity of *Leishmania* spp.–positive goats ranked first. Although our data do not necessarily mean that goats constitute a reservoir host of *L. donovani*, these observations indicate the need for further investigation of goats’ possible role in VL transmission.

## Abstract 2

Proximity of *Leishmania donovani*–positive goats is a risk factor for human infection.

Visceral leishmaniasis (VL), also known as kala-azar, is a fatal vector-borne parasitic disease. Worldwide incidence is 500,000 cases per year; ≈90% of cases occur in India, Nepal, Bangladesh, Sudan, and Brazil (*1*). On the Indian subcontinent, the number of officially reported cases, although only a fraction of the true incidence (*2*), has increased during the past 5–6 years, and the disease is spreading to new areas (*3*). A kala-azar elimination program was recently launched by the governments of Bangladesh, India, and Nepal, with the support of the World Health Organization; the goal is to reduce the annual incidence in VL-in endemic regions to <1 case per 10,000 persons by 2015 (*4*). The program essentially relies on early diagnosis and treatment of persons and on vector control (*5*). This strategy is based on the assumption that *Leishmania donovani*, the etiologic agent of VL, is transmitted from person to person (anthroponotic VL).

The possible role of domestic animals in anthroponotic VL has been studied in Bangladesh (*6*), but no clear conclusions have been drawn with regard to animals as risk factors or reservoir hosts. In contrast, the proximity to a VL-infected person is a major risk factor for VL (*6*). Thus, persons are still considered the only reservoir host for *L. donovani* on the Indian subcontinent. However, the reasons for persistence during interepidemic periods are debated, and dermal leishmaniasis after kala-azar has been incriminated (*7*). Correct identification of the *Leishmania* reservoir host is crucial for the design of control programs. Molecular tools offer new opportunities to better document and reassess transmission patterns. To explore the potential role of domestic animals in transmission, we performed an extensive study in an area of active VL transmission in Nepal, mapping *Leishmania* infections among healthy persons and domestic animals.

## Materials and Methods

### Study Site

**Figure 1:**
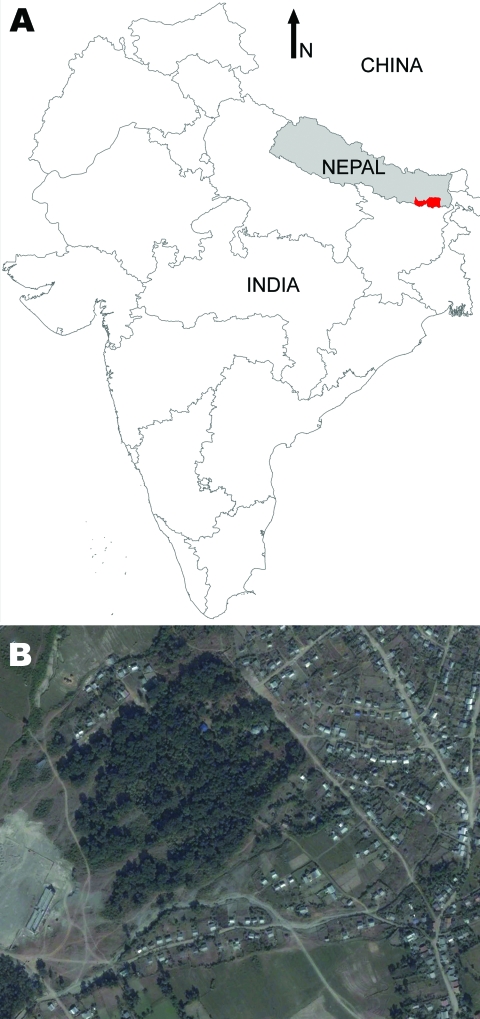
A) Visceral leishmaniasis–endemic area (red) of Nepal under study by KALANET project (www.kalanetproject.org); B) satellite picture of Dharan-17, Nepal. Copyrights 2009 Google Image; 2009 DigitalGlobe; 2009 Europa Technologies; and 2009 Mapabc.com.

**Figure 2:**
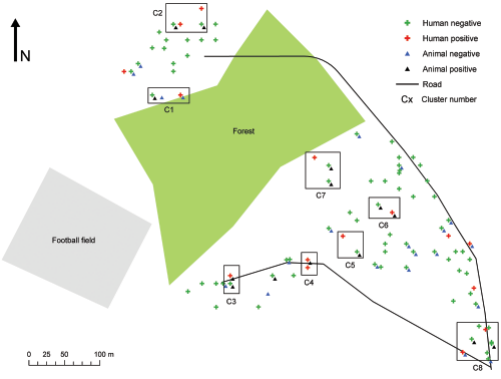
Distribution of sampled households and domestic animals, by visceral leishmaniasis status as determined by PCR, Dharan-17, Nepal, September 2007–February 2008.

The study was conducted as part of the KALANET project, a community trial of insecticide-treated bed nets (www.kalanetproject.org) in the Terai region of eastern Nepal. In Nepal, each village is divided into several wards. For the KALANET project, 10 wards with active VL transmission were selected. Dharan-17 was 1 such ward; it is a periurban ward in the Dharan municipality, located in the foothills of the Mahabharata hills and along the bank of Sardu River. Dharan-17 covers ≈0.3 km^2^ (Figure 1) and has 515 inhabitants living in 105 households (Figure 2). A demographic survey conducted in July 2006 showed that 77% of households had at least 1 domestic animal (i.e., cow, goat, dog). Most cows, buffaloes, and goats were kept <10 m from the households at night, although a few goats were kept inside the house. VL was only recently reported in this periurban area; Dharan-17 has an average VL incidence rate of 1.61% per year (for 2004–2006). Furthermore, during a previous study conducted in 2006, we documented a higher rate of VL positivity by PCR among the healthy persons in Dharan-17 compared with those in the 9 other wards in the KALANET trial, possibly suggesting a high transmission rate (*8*). For a control area, we selected Dhankura-3 in Patlekhola. This ward is ≈60 km from Dharan-17, in a hilly area where no VL cases have yet been reported.

### Ethical Aspects

Ethical clearance for the KALANET project was obtained from the Ethical Committee of the B.P. Koirala Institute of Health Sciences (BPKIHS), Dharan, Nepal, and the corresponding bodies at the Institute of Tropical Medicine, Antwerp, Belgium, and the London School of Hygiene and Tropical Medicine, London, UK. A community meeting informed local leaders and village residents about the study purpose; informed consent was obtained from all animal owners before their animals were included in the study. International animal experimentation guidelines were followed. Persons provided written consent before enrollment and providing blood samples, per the human experimentation guidelines approved by BPKIHS and the corresponding body at the Institute of Tropical Medicine, Antwerp. For religious reasons, 10.67% of persons did not provide written consent to donate their blood samples.

### Sample Collection

All animal surveys were conducted by experienced veterinarians. In Dharan-17, a house-to-house survey was first conducted in September 2007, among 105 households, to collect information on the number and types of animals present in the ward; only information about bovines (cows and buffaloes) and goats was collected in this first survey. Later, 2 sampling surveys were conducted in this ward. In October 2007, survey I sampled 144 goats, 24 buffaloes, and 20 cows from the 37 households that had >1 bovine or goat. In February 2008, survey II focused on 6 households in which *Leishmania* spp.–positive animals had been identified during survey I. Although the owners claimed that the goats in surveys I and II were the same goats, we could not confirm this. In February 2008, samples were collected from 25 goats, 17 buffaloes, and 21 cows in the control area. In addition to animal samples, we also collected 278 blood samples from persons, all >5 years of age, who lived in Dharan-17 at the time of the survey and provided consent. The samples (1 mL) were collected by venipuncture from animals and persons into tubes containing molecular biology grade Na_2_EDTA (240 μg/mL of blood; Sigma-Aldrich, Bornem, Belgium). All tubes were immediately stored in a chilled ice box and transferred on the same day to the laboratory at BPKIHS, where 180 μL of each sample was transferred to a tube containing 180 μL of AS1 buffer (catalog no. 1006243; QIAGEN, Venlo, the Netherlands), mixed well, and stored at room temperature.

### DNA Extraction and PCR Amplification

All blood samples stored in AS1 buffer were used to extract the DNA within 1 month. The QIAamp DNA Mini Kit (catalog no. 56301; QIAGEN) was used to extract DNA at BPKIHS, following manufacturer’s instructions. All DNA samples were sent at ambient temperature to the Institute of Tropical Medicine, Antwerp, where they were analyzed by PCR specific for small ribosomal genes of *Leishmania* spp. as described elsewhere (*9*). To confirm that the amplified DNA corresponded to *Leishmania* spp., amplicons from a set of positive samples were sequenced. The sequences were compared with those of *Leishmania* spp. and other trypanosomatids from GenBank.

### Spatial Clustering of *Leishmania* spp.–positive Households

We used the results from survey I to assess the clustering of the *Leishmania* spp.–positive samples in Dharan-17. Each household, previously georeferenced by a geographic positioning system and mapped by using ArcGIS 9.2 (ESRI, Redlands, CA, USA), was identified as *Leishmania* spp.–positive or –negative for animal and human samples. Analyses considered all animals (goats, cows, and buffaloes) together. The bivariate K-function was used to determine whether households with *Leishmania* spp.–positive persons were spatially clustered around households with *Leishmania* spp.–positive domestic animals in Dharan-17. The following equation was used (*10*): K(*d*) = expected no. events B within distance *d* of arbitrary event A / overall density of events B. For easier interpretation of the results, the bivariate K-function was transformed in an L-function as follows (*11*):


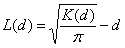


Positive L(*d*) results would suggest that human and animal *Leishmania* spp.– positive households are spatially associated. Further technical details on the bivariate K-function are available in the Technical Appendix(*10*–*12*).

### Classification Trees

Classification trees (CTs) and regression trees (Salford Systems, San Diego, CA, USA) can be used in classification and regression problems (*13*). We used CTs to analyze risk factors and identify interactions for *Leishmania* spp.–positive households. This nonlinear, assumption-free, and algorithm-based method splits data variance across nested nodes (with increased importance toward the tree base). Its algorithm automatically eliminates variables without explanatory power. Sensitivity (% true positives) and specificity (% true negatives) were computed. A 10-fold cross-validation (leaving 10% of the data at a time for the whole dataset to compute percentage of misclassifications) was used. Node impurity was measured by using the Gini index, and the minimal size of the base and terminal nodes were set to 10 and 1 cases, respectively. The method is explained in more detail elsewhere (Technical Appendix) (*14*–*17*). The factors included in the CT analysis were 1) household (i.e., type of house, head of household occupation/education) and biologic (i.e., density of domestic animals around households, collected in a house-to-house survey in July 2006 and estimated by using a kernel density function [ArcGIS, ESRI]) (*6*) and 2) results of PCR analyses (Table 1).

**Table 1:** Risk factors included in classification tree analysis of visceral leishmaniasis in Nepal*

Biological factors (animals)
No./household
Samples from buffalo, cow, or goat
*Leishmania* spp.–negative samples from buffalo, cow, or goat
No. households with *Leishmania* spp.–positive animals 10, 25, 50, or 100 m from house
Minimum, mean, and maximum distance to household with *Leishmania* spp.–negative animal
Nos. poultry, birds, poultry and birds, pigs, dogs
Density (no./km^2^) of poultry, birds, poultry and birds, buffaloes, cows, goats, pigs
Biological factors (humans)
No./household
Samples for PCR
*Leishmania* spp.–negative samples
No. households with *Leishmania* spp.–positive persons 10, 25, 50, or 100 m from house
Dichotomous variable for positive households for humans
Minimum, mean, and maximum distance to household with *Leishmania* spp.–positive person
Household factors
No. houses, identity of household, latitude, longitude, education of head of household, occupation of head of household, category house type, socioeconomic index
No. persons/ household, rooms, persons/room, bed nets, bed nets/person

*More details available upon request from J.-C.D.

## Results

### Animal Sample PCR Results

Survey I found 188 domestic animals (goats, buffaloes, and cows) in only 37 of the 105 households of Dharan-17. The overall rate of *Leishmania* positivity was 13.3% (25/188); goats accounted for 16% (23/144), cows for 5% (1/20), and buffaloes for 4% (1/24).

The *Leishmania* spp.–positive animals were encountered in the 15 households shown in Figure 2. During survey II, only goats were sampled from 6 of these households; 2 goats (from different households) of 24 (8.34%) were *Leishmania* spp. positive. All sequenced amplicons confirmed the presence of a *Leishmania-*specific sequence. All 63 samples from animals in the control area were negative.

### Human Sample PCR Results and Spatial Clustering

In Dharan-17, of the 278 persons sampled, 17 (6.1%) were *Leishmania* spp. positive, 14 were healthy with no history of kala-azar, and none was a household contact of a VL case-patient. Of the 17 *Leishmania* spp.–positive persons, 2 had had VL and had been successfully treated. We could not determine the history for 1 person because he had moved out of the ward. The 17 *Leishmania* spp.–positive persons were from 16 households. The Kuldorff spatial scan statistic (*18*) was used to assess whether *Leishmania* spp.–positive persons were clustered in Dharan-17, but no significant clusters were detected. Analogous results were obtained for *Leishmania* spp.–positive animals (results not shown). However, when we superimposed households and *Leishmania* spp.–positive persons or animals (Figure 2), it visually appeared that 1) in 8 sites of the ward, *Leishmania* spp.–positive persons were localized in households near where *Leishmania* spp.–positive animals were kept (distance between the households <30 m; further called clusters), and that 2) in 1 site only *Leishmania* spp.–positive persons were found (Table 2). Some clusters constituted hot spots (had several cases of infection): clusters 2 (9 animals and 2 persons) and 8 (6 animals and 2 persons). The clusters represented in Figure 2 and detailed in Table 2 were determined visually; no statistical methods were applied. The bivariate K-function results show that households with *Leishmania* spp.–positive persons were clustered around households with *Leishmania* spp.–positive animals; *L*(*d*) is positive from 0 to 100 m. However, the spatial association between them is only significant from 0 to 5 m (Technical Appendix).

**Table 2:** Distribution of *Leishmania* spp.–negative cases of visceral leishmaniasis among clusters, Nepal, September 2007–February 2008*

Cluster	No. animals	No. humans
C1	1	1
C2	9	2
C3	2	1
C4	1	4
C5	1	1
C6	3	1
C7	3	1
C8	6	2
Out of cluster	1	4

*Cluster group of households situated near (<30 m) each other and in which *Leishmania* spp.–negative animals or humans were encountered.

### Classification Tree Analysis

**Figure 3:**
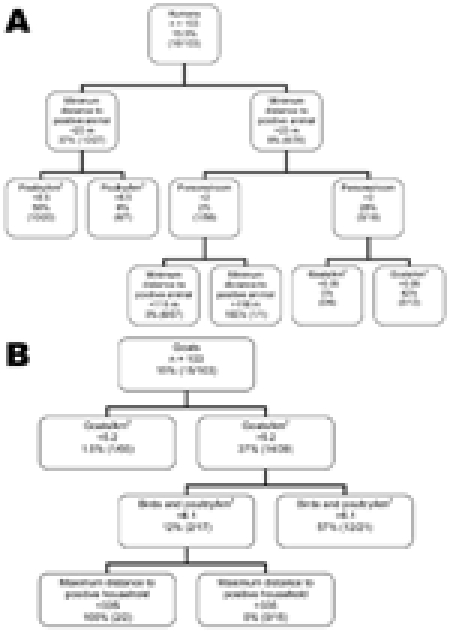
Classification tree results, showing interplay between risk factors of *Leishmania* positivity, determined by PCR, for A) humans and B) goats, in Dharan-17, Nepal, September 2007–February 2008.

The bivariate K-function analyzes only the relationship between *Leishmania* spp.–positive households for persons and domestic animals in Dharan. The results of this function have no meaning other than a spatial grouping of households with positive animals and persons. In a second stage, we used a CT model to analyze risk factors for *Leishmania* spp.–positive households. First we analyzed households in which *Leishmania* spp.–positive persons had been encountered. These results showed that the minimum distance to a household with a *Leishmania* spp.–positive animal (any species) was the variable with the highest discriminatory power. It appears first in the tree (Figure 3, panel A) and gets a relative importance score of 100% (data not shown). Discriminating distance was 22.8 m: households <22.8 m from a household with *Leishmania* spp.–positive animals showed a 37% probability of hosting *Leishmania* spp.–positive persons versus 7.9% if they were >22.8 m from a household with *Leishmania* spp.–positive animals. The next variables appearing on the tree were the density in poultry (higher risk for *Leishmania* spp.–positive persons if density <8.55/km^2^), the number of persons per room (higher risk if >2.88 persons/room), and density of goats (higher risk if >0.05 goats/ km^2^). A second CT analysis was conducted for households in which *Leishmania* spp.–positive animals had been encountered. The generated tree differed from the previous one in that the first discriminating variable was the density of goats per km^2^; risk for *Leishmania* spp. positivity associated with a density >5.2 (36.8% vs. 1.5%; Figure 3, panel B) was higher, and a relative importance score was 100% (data not shown). The next variables appearing on the tree were the density of birds and poultry per km^2^ (higher risk for *Leishmania* spp.–positive goats if >6.14) and the maximum distance to a household with a *Leishmania* spp.–positive person (higher risk if <334.6 m). All these variables also appeared with highest predictor ranking scores (data not shown).

For the tree in Figure 3, panel A, the sensitivity and specificity of the tree were 100% and 80.5%, respectively. For the tree in Figure 3, panel B, sensitivity and specificity were 93.3% and 89.8%, respectively.

The minimum distance to a positive animal split in Figure 3, panel A, is difficult to interpret. It involves 1 positive result and is under a branch that was determined to be predominantly negative as a result of the first split when using the same variable at >23 m. This split should therefore be interpreted with caution. The same applies for the split of maximum distance to positive household <335 m (Figure 3, panel B), which is difficult to interpret if the study area is only 300 m^2^ and may be the result of the irregular shape of the study area.

## Discussion

We found *Leishmania* DNA in domestic animals (cows, buffaloes, and goats) from Dharan-17, mostly in goats (16%), although no goats or other animals in the control area were *Leishmania* spp. positive. As DNA persists in the body for only a short time (24 h) (*19*), PCR positivity is a good indicator of current (or recent) infection. Considering the time between the *Leishmania* spp. peak transmission season in Nepal (estimated in April–May) (*20*) and our first survey (October 2007), our results thus indicate that goats can be infected with *L. donovani* and that this infection persists for at least several months. Contact with *Leishmania* organisms, as shown by serologic findings, has already been reported for goats and other domestic animals in Sudan (*21*).

The decrease in *Leishmania* spp. positivity between the 2 surveys (from 16% in October 2007 to 8.34% in February 2008) could be explained by several reasons: 1) sampling bias, 2) effective decrease of parasitemia over a certain period because of immunologic control of infection, or 3) disappearance of positive animals from the ward. A follow-up study of *Leishmania* spp.–infected goats (up to at least 12 months) combined with an adequate tracking system are needed to determine whether parasites are still in the blood of the animals during the next transmission peak.

Comparison of the results from animals with those from healthy human volunteers from Dharan-17 provided additional interpretation of the animal results. *Leishmania* spp. positivity was found to be ≈3× lower among persons than among animals (6.1% vs. 16%, respectively). These data are consistent with data on the feeding behavior of *Phlebotomus argentipes* blood-sucking flies, reported previously (*22*). This species seems to breed essentially in cattle sheds (*23*) and are 5× more attracted to cattle than to persons (*24*,*25*) and feed more on animals (62.80%) than on persons (24.92%), according to a study in India (*22*).

Information about the association of PCR results for persons and domestic animals can be used to investigate the role of domestic animals in *L. donovani* transmission. Visual inspection of the data suggested that most of the *Leishmania* spp.–positive persons were living near *Leishmania* spp.–positive goats. This observation was confirmed by bivariate K-function results and CT analysis. Distance of clustering between *Leishmania* spp.–positive persons and *Leishmania* spp.–positive domestic animals varied slightly according to the method (K-function up to 5 m; CT analysis <22.8 m) and was less than the flight range of *P. argentipes* flies. Differences could be explained by the inclusion of *Leishmania* spp.–negative households in the CT analysis. The 2 types of CT analyses pointed more to the role of biologic factors than to household factors like education, bed-net use, or type of house. Additional studies should explore the presence of poultry as a risk factor, as has been reported for urban VL in Brazil (*26*).

Even if our results indicate that goats might be involved in the dynamics of VL, they do not necessarily mean that these animals constitute a reservoir host for *L. donovani*. Criteria for the definition of *Leishmania* reservoir hosts were recently reviewed (*27*) and include sandfly foraging behavior and feeding preferences and the dynamics of infections in assumed reservoir hosts; a key question is the clearance times (chronicity) of infections. Whether the phenomenon observed here can be extrapolated to other VL-endemic foci should also be explored. Dharan-17 is a new emerging focus, and in the absence of immunity, human and animal populations could be more sensitive to *Leishmania* infections. Our observations warrant further investigation and a close monitoring of goats and other peridomiciliary animals like rodents and birds. If the role of these animals in the transmission cycle is confirmed, the potential implications could affect VL control programs in the region.

## Supplementary Material

Technical Appendix Domestic Animals and Epidemiology of Visceral Leishmaniasis, Nepal
