# Supplementary material for: Domestic Animals and Epidemiology of Visceral Leishmaniasis, Nepal
Source: Emerg Infect Dis. 2010 Feb;16(2):231–7. doi: 10.3201/eid1602.090623 (PMC2958000; doi:10.3201/eid1602.090623)
Supplement: Technical Appendix — Domestic Animals and Epidemiology of Visceral Leishmaniasis, Nepal [file 09-0623_Techapp-s1.pdf]

Domestic Animals and Epidemiology of Visceral Leishmaniasis, Nepal

K-function

Materials and Methods

The bivariate (cross) K-function is an extension of the Ripley K-function, which is a method to test for global clustering in a point pattern in a planar space. The bivariate K-function estimates the spatial dependence between 2 events located in a defined area (i.e., village) by measuring the expected number of events B within a distance *d* of an arbitrary event A, divided by the overall density of the events B (equation 1) (*1*).

$$K(d) = \frac{\text{expected no. events B within a distance } d \text{ of an arbitrary event A}}{\text{Overall density of events B}} \qquad (1)$$

To provide an easy linear interpretation of the interactions among human and animal *Leishmania*-positive households, we represented the bivariate K-function results as an L-function (equation 2) (*2*).

$$L(d) = \sqrt{\frac{K(d)}{\pi}} - d \quad (2)$$

If human and animal *Leishmania*-positive households are spatially independent, the expected value of L(*d*) is zero. In the present study L(*d*) was calculated at 5 m increment distance bands, the human *Leishmania*-positive households are the events A in equation 1 whereas the animal *Leishmania*-positive households are the events B. The statistical significance of the L(*d*) results was assessed by comparing them with the 95% confidence envelopes of the bivariate function estimated from 10,000 simulations assuming a random distribution of *Leishmania*-positive households. The random toroidal shifts method applied determined the confidence bands by randomly shifting the locations of animal *Leishmania*-positive households. The bivariate K-function applied did not include a formal edge correction, but the study area considered in the K-function calculations was enlarged to minimize the edge effect. The bivariate K-function was calculated by using the package Splancs in R (*3*,*4*).

Results

Bivariate k-function results presented in the Figure show that human *Leishmania*-positive households tend to be clustered around animal *Leishmania*-positive households in Dharan-17. However the spatial association is only statistically significant between 0 and 5 m.

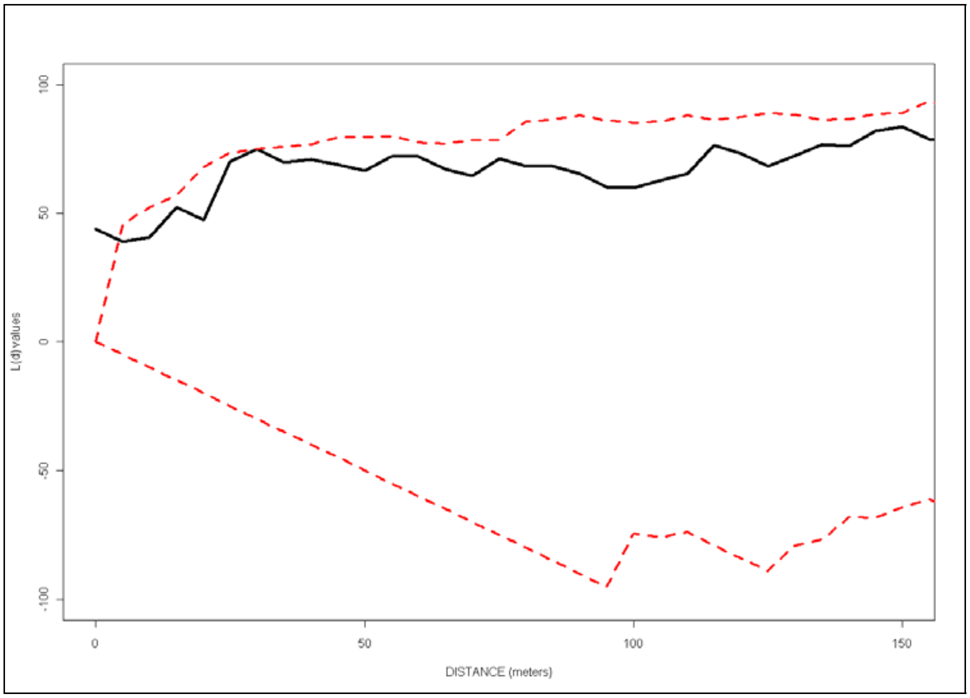

Figure. Bivariate L-function (L(*d*)) plot to evaluate if human *Leishmania*-positive households are clustered around domestic animal *Leishmania*-positive households in Dharan-17. Solid line is the plot of L(*d*) for *Leishmania*-positive households. Red dotted lines are the 95% confidence envelopes of L(*d*) estimated from 10,000 simulations (random toroidal shifts).

Classification trees

Classification trees provide a predictor ranking based on the contribution predictors make to the construction of the tree. This indicates how important the different independent variables are in determining the division of individuals into infected and noninfected nodes. It is possible that a variable never enters the tree as the splitter but would have been a second best at different points in the tree building. Such a “surrogate” splitter will turn out as very important in the variable importance ranking provided by CART because the system memorizes this into the global variable importance score. It can be noted that the more basal a node is, the more “variability” or “useful information” is present. The lack of assumptions regarding spatial

independence of variables in comparison to more traditional approaches like logistic regression is a further advantage of the method at hand.

Model specifications were as follows:

a. Criteria used for predictive accuracy (% misclassified or variance): The sensitivity and specificity of the tree were computed. The sensitivity is defined as follows: Here, sensitivity means the probability that a positive case is correctly identified as a positive case and specificity means the probability that a negative case is correctly identified as a negative case (*5*).

b. Impurity measures: As an impurity measure the Gini index was used. For classification problems, decision trees give the user the choice of several impurity measures: The Gini index, Chi-square, or G-square. The Gini index of node impurity is the measure most commonly chosen for classification-type problems.

c. Stopping rules: The Terminal node and Parental node minimal cases were set to be 1 and 10, respectively.

d. Estimation of accuracy (node impurity). The Gini index was used as a measure of impurity for the nodes.

e. Cross validation measures used: A 10-fold cross validation was used.

References

1. Lotwick HW, Silverman BW. Methods for analysing spatial processes of several types of points. J R Stat Soc [Ser A]. 1982;B44:406–13.

2. Bailey TC, Gatrell AC. Interactive spatial data analysis. John Wiley & Sons, New York, NY.1995.

3. Rowlingson BS, Diggle PJ. SLANCS: spatial point pattern analysis code in S-PLUS. Comput Geosci. 1993;19:627–55. DOI: 10.1016/0098-3004(93)90099-Q

4. Bivand R, Gebhardt A. Implementing functions for spatial statistical analysis using R language. J Geogr Syst. 2000;2:307–17. DOI: 10.1007/PL00011460

5. Cook EF, Goldman L. Empiric comparison of multivariate analytic techniques: advantages and disadvantages of recursive partitioning analysis. J Chronic Dis. 1984;37:721–31. PubMed DOI: 10.1016/0021-9681(84)90041-9
